# Diabetes mellitus and periodontal disease: awareness and practice among doctors working in public general out-patient clinics in Kowloon West Cluster of Hong Kong

**DOI:** 10.1186/s12875-018-0887-2

**Published:** 2018-12-17

**Authors:** Sut Yee Tse

**Affiliations:** Department of Family Medicine and Primary Health Care, New Territories West Cluster, Hospital Authority, Hong Kong, China

**Keywords:** Awareness, Practice, Diabetes, Periodontal disease

## Abstract

**Background:**

Diabetes mellitus (DM) and periodontitis are very common and they interact with each other bidirectionally. This survey studied public primary care doctors on their awareness of this bidirectional relationship and their corresponding practice.

**Methods:**

All Family Medicine doctors in Kowloon West Cluster, Hospital Authority were invited to a cross-sectional questionnaire survey. Results were analyzed statistically.

**Results:**

One hundred sixty-eight questionnaires were sent out, 143 were returned (response rate 85.1%). One hundred forty valid questionnaires were analyzed. Ninety-two percent of participants were aware of a relationship between DM and periodontal disease and this awareness was not associated with their years of experience, training status and personal oral health behavior. Ninety percent knew the effect of poor DM control on periodontal disease but only 76% were aware of the reverse effect of periodontal disease on DM. The difference was statistically significant (*p* = 0.002, Related-samples Sign Test). In clinical practice on DM patients, only 5.7% asked dental history often (defined as 50% patients or above), 7.1% examined their mouths often and 12.1% recommended them to see dentist often. Logistic regression showed that awareness factors had no association with periodontology related clinical practice whereas clinical experience, being a Family Medicine specialist and personal interdental cleaning habit were linked with more positive practice.

**Conclusions:**

A high proportion of doctors in the study were aware of the relationship between DM and periodontal disease. However, this did not appear to influence their practice. Further measures among doctors and patients to promote comprehensive management of DM and periodontal disease should be explored.

**Electronic supplementary material:**

The online version of this article (10.1186/s12875-018-0887-2) contains supplementary material, which is available to authorized users.

## Background

Diabetes mellitus (DM) is a chronic metabolic disease characterized by hyperglycemia secondary to dysregulated insulin activity. It is a major cause of blindness, kidney failure, heart attack, stroke and lower limb amputation. Periodontal disease or periodontitis is a chronic oral infection that results in loss of periodontal attachment, bone destruction and eventually the loss of teeth. Features of periodontal disease may include swollen and bleeding gum, gum recession, gum pocket formation, bad breath and loose teeth.

DM is widely accepted as a major risk factor for periodontal disease. One study showed that patients with DM had approximately three-fold increased risk in developing periodontal disease [1]. Another study showed a significantly higher prevalence of periodontal disease in patients with type 2 DM when compared with non-DM controls (50% versus 36%) [2]. Besides prevalence, DM also affects the severity and progression of periodontal disease [3]. Patients with poorly controlled DM may have higher risk of developing severe periodontal disease when compared with non-DM controls [4]. They also have more severe gingival bleeding and higher level of gingival inflammation than those with well controlled DM or non-DM controls [5, 6]. Poorly controlled DM patients also have more advanced alveolar bone loss [7, 8] and periodontal destruction [9, 10], worse outcome following periodontal treatment [11], more recurrent periodontal infection and less favorable long term prognosis [12]. Periodontal disease is therefore regarded as the sixth complication of DM [13].

Conversely, periodontal disease may worsen the glucose control in DM patients. There is growing evidence to suggest that the relationship between DM and periodontal disease is bidirectional [14–16]. Periodontal disease has been shown to adversely affect glucose control in DM patients and also increase the risk of developing DM complications [17]. DM patients with severe periodontal disease are more prone to other DM complications and have a higher mortality rate than those DM patients without severe periodontal disease [18]. Moreover, effective periodontal treatment appears to improve DM control. Patients with type 2 DM receiving comprehensive dental treatment is associated with improvement in their glucose control with reduction of 0.9% in HbA1c level when compared with those DM patients without dental treatment [19]. Provided that the HbA1c reduction could be sustained, it would translate into a reduction of DM complications and mortality [20].

Both DM and periodontal disease are prevalent. The World Health Organization reported that in 2014, globally 8.5% of adults aged 18 years and above had DM [21] while 10–15% of the world populations suffered from severe periodontitis [22].

In Hong Kong, the prevalence of both diseases was even higher. Locally, the prevalence of type 2 DM was about 10% [23]. According to the Oral Health Survey 2011 in Hong Kong, about 40% adults and 60% of non-institutionalized elderly had some degree of periodontal disease [24, 25].

A large proportion of DM patients in Hong Kong are managed in General Out-Patient Clinics (GOPCs) which belong to the public sector. According to Hospital Authority (HA) internal statistics 2016, about 35% of all DM patients in Hong Kong are managed in GOPCs and Kowloon West Cluster (KWC) GOPCs are responsible for ¼ of them. Dental service in Hong Kong is mainly provided by private dentists and hence majority of patients with periodontal disease are managed in the private sector.

It is expected that if doctors know that two conditions are associated, when treating either one condition, they would look for the other condition and hence leading to better practice. However this may not be always true. Literature showed that better awareness might or might not be associated with better practice. In a study on primary care physicians in Spain, better knowledge of the physicians on antibiotics use was shown to be associated with more appropriate prescription of antibiotics [26]. Conversely in another study on residents of various specialties in an Iranian hospital, though the residents’ knowledge and awareness on hand hygiene was satisfactory, they had poor hand hygiene practice [27].

This study aimed to answer the two research questions: 1) were our public primary care doctors aware of the bidirectional relationship between DM and periodontal disease? 2) if they were aware of the relationship, did this influence their daily practice? Doctors working in the Department of Family Medicine and Primary Health Care in KWC of HA were chosen for the study. At the time of the study, this department was the largest among the 7 Family Medicine departments in HA. It had the largest number of doctors and clinics and was responsible for the management of ¼ of all DM patients attending GOPCs in Hong Kong.

### Objectives

- To determine the proportion of the surveyed doctors who were aware of the interrelationship between DM and periodontal disease;
- To understand the practice of the surveyed doctors in relation to periodontal disease when managing DM patients.

## Method

### Study design and measurements

Doctors’ awareness and practice regarding DM and periodontal disease was assessed by a cross-sectional questionnaire survey. There was no validated questionnaire on this aspect suitable for local use. Thus, a tailor-made questionnaire was developed for this study [28]. To establish face and content validity, the questionnaire was designed by the author in collaboration with a professor of periodontology in the Faculty of Dentistry, the University of Hong Kong. Then the draft questionnaire was critiqued by an expert panel comprising two endocrinologists, two family medicine (FM) specialists and a periodontologist. Each question was evaluated by the panel whether it was essential, useful or irrelevant to achieving the study objectives. The accuracy and clarity of the questionnaire was also commented and improved by the expert panel. The questionnaire was then finalized after collecting all the comments from the expert panel. Together with the periodontology professor who assisted in designing the questionnaire, the final questionnaire had passed the appraisal of two experts in each corresponding field.

The questionnaire (Additional file 1) was divided into three parts. The first part assessed doctors’ awareness of the relationship between DM and periodontal disease and the source they obtained such information from. The second part enquired doctors’ relevant oral health practice when managing DM patients. The third part asked the doctors for their demography, job-related information and personal oral health behavior.

Then, face to face cognitive debriefing was conducted on 5 doctors with similar background and characteristics of the target participants. They were interviewed by the principal investigator for their understanding on individual question. The questionnaire was modified afterwards to improve the clarity of the questions.

After that, a pilot study was conducted on 20 primary care doctors with similar background and demography of the target participants. They were asked to do the questionnaire twice separated by 2 weeks interval. Intraclass correlation coefficient (ICC) and Cronbach’s alpha were calculated on the two sets of results to assess the test-retest reliability and internal consistency.

The questionnaire together with a cover letter were distributed by doctor-in-charge of each clinic to each participant. Participation in the study was voluntary and anonymous. Consent to participate in the study was implied if the doctor returned the completed questionnaire voluntarily. Completed questionnaires were returned to the author within 2 weeks using confidential envelopes. Emails were sent 1 week before the deadline to remind participants to return the questionnaire.

### Subjects

All 168 doctors in 23 GOPCs of the Department of Family Medicine and Primary Health Care in KWC were included in the study.

### Study outcomes

- **Primary Outcome**
  - the proportion of the surveyed doctors who were aware of the interrelationship between DM and periodontal disease and the proportion of their different practice in relation to periodontal disease when managing DM patients.
- **Secondary Outcome**
  - the source of information of the surveyed doctors on the relationship between DM and periodontal disease;
  - the interrelationships among *awareness* of DM and periodontal disease, corresponding clinical *practice*, *demography* and *personal oral health behavior* data of the surveyed doctors.

### Statistical methods

#### Sample size estimation

There were 168 doctors in the target population. The study aimed at studying all of them. However in order to achieve 95% confidence level and not more than 5% margin of error, at least 118 of them had to be sampled assuming the most demanding statistical scenario of 50% response distribution [29] http://www.raosoft.com/samplesize.html.

#### Data analysis

As there was no gold standard or established scoring system in this topic, the results of the questionnaire were analyzed by descriptive statistics. Participants’ demographic data, personal oral health behavior, awareness and practice data were presented in frequency distribution tables or charts. Pearson chi-square tests/ Fisher’s exact tests for the association of the awareness questions result with demographic and personal oral health behavior data were performed. Difference between the proportion of participants knowing DM-to-periodontitis and periodontitis-to-DM relationships was analyzed by Related-samples Sign Test. Bivariate logistic regression was conducted to explore factors that might associate with doctors’ practice.

Sample size estimation before the study was performed by on-line sample size calculator (http://www.raosoft.com/samplesize.html). Data analysis was done with the Statistical Package for the Social Sciences (IBM SPSS Statistics) version 22. Summary graphics were produced from Microsoft Excel 2010. *P*-value less than 0.05 was considered statistically significant. Missing data were handled in listwise deletion approach.

## Results

### Pilot study

Pilot study on 20 primary care doctors showed that the ICC was 0.760 (95% confidence interval 0.580–0.888). Cronbach’s alpha for part 1 (awareness) and part 2 (practice) of the questionnaire was 0.720 and 0.884 respectively.

### Main study

The Main Study was conducted shortly after the pilot study. A total of 168 questionnaires were sent out. One hundred forty-three questionnaires were returned (response rate 85.1%). Three of them were discarded due to incomplete answers. Thus, 140 (83.3%) valid questionnaires were analyzed.

The demographic data of the participants and their oral health behavior were summarized in Table 1. There were slightly more male than female among the participants. Their age ranged from 24 to 71 with a mean of 37.25 (SD 9.35) years. Their mean years of experience was 13.14 (SD 8.66) years. 40.8% were FM trainees (including both basic and higher trainees). FM specialist to trainee to other doctor ratios were roughly 2:2:1. Majority had regular dental checkup within recent 1 year. 91.4% brushed twice or more than twice a day and more than 40% practiced interdental cleaning daily.

**Table 1:** Demographic and personal oral health behavior data of the participants (*n* = 140)

Demography/background	Number (percentage)
Gender	
Male	75 (53.6%)
Female	65 (46.4%)
Age (in years)	
≤29	30 (21.4%)
30–39	63 (45.0%)
40–49	32 (22.9%)
50–59	11 (7.9%)
≥60	4 (2.9%)
Years after graduation	
0–5	29 (20.7%)
6–10	30 (21.4%)
11–15	36 (25.7%)
16–20	20 (14.3%)
> 20	25 (17.9%)
Place of graduation for primary medical degree	
Hong Kong	123 (87.9%)
Mainland China	9 (6.4%)
Australia	6 (4.3%)
Others	2 (1.4%)^a^
Working status	
Part time	9 (6.4%)
Full time	131 (93.6%)
Training status^b^	
Basic trainee	39 (27.9%)
Higher trainee	18 (12.9%)
FM specialist	53 (37.9%)
Non-trainee, non-specialist	30 (21.4%)
Personal oral health behavior	
Last attend dentist	
Within 1 year, regular check up	85 (60.7%)
Within 1 year, irregular check up	15 (10.7%)
More than 3 years	9 (6.4%)
Daily tooth brushing	
More than twice	16 (11.4%)
Twice	112 (80.0%)
Once	12 (6.6%)
Interdental cleaning	
Everyday	59 (42.1%)
Occasional	67 (47.9%)
Never	14 (10.0%)

^a^one from Taiwan, one from Rangoon

^b^Family Medicine training in Hong Kong consists of 4 years (minimum) of basic training and 2 years (minimum) of higher training. The 4 years of basic training is composed of 2 years of hospital based and 2 years of community based training. Then the trainee has to pass the conjoint HKCFP/RACGP fellowship examination before he/she can be enrolled to the higher training. Upon completion of the higher training, the trainee needs to pass the exit examination before being qualified as a Family Medicine specialist

Results of the awareness of the relationship between DM and periodontal disease were summarized in Table 2. Majority of them were aware that there was a relationship in general (Question 1 - Q1). A series of Pearson chi-square tests were done to explore if there was any relationship between this awareness (Q1–5, independently) and participants’ demography and oral health behavior. No association between them was found.

**Table 2:** Awareness of relationship between DM and periodontal disease

Awareness questions	Agree	Uncertain	Disagree
General awareness			
Q1 Relationship exists between DM and poor oral health.	129 (92.1%)	9 (6.4%)	2 (1.4%)
Awareness of DM influencing periodontal health			
Q2 Periodontal disease (periodontitis) is one of the DM complications.	108 (77.1%)	28 (20.0%)	4 (2.9%)
Q4 Patients with poor glycemic control are at higher risk of developing periodontitis.	126 (90.0%)	13 (9.3%)	1 (7.0%)
Awareness of periodontal disease affecting DM			
Q3 Poor periodontal/gum health may be associated with more difficult control of blood glucose level in DM patients.	106 (75.7%)	28 (20.0%)	6 (4.3%)
Q5 Treatment of gum/periodontal disease may be associated with improved glycemic control of DM patients.	85 (60.7%)	48 (34.3%)	7 (5.0%)
Awareness concerning proper periodontal management of DM patient			
Q6 Treatment of gum/periodontal disease involves regular visits to dentist for checkup and treatment as well as proper daily oral hygiene care.	137 (97.9%)	3 (2.1%)	0 (0.0%)
Q7 Which of the following tools is/are essential for daily oral hygiene care in DM patients? (only toothbrush AND floss/interdental brush TOGETHER with NO OTHER CHOICE is considered correct)	Toothbrush 139 (99.3%)		Only 93 (66.4%) answer correctly
	Floss/interdental brush 130 (92.9%)		
	Toothpick 6 (4.3%)		
	Mouthwash 41 (29.3%)		

More specifically, 77.1% participants knew that periodontal disease was a complication of DM (Q2); and 90% participants knew the effect of poor DM control on periodontal disease (Q4). Conversely, the effect of poor periodontal disease on DM control was known by 75.7% of the surveyed (Q3). The difference between Q4 and Q3 (90% vs 75.7%) was statistically significant (*p* = 0.002, Related-samples Sign Test). There was no statistical evidence to indicate that if a participant knew the DM-to-periodontitis relationship, he/she was more likely (or unlikely) to know the reverse periodontitis-to-DM relationship, and vice versa (*p* = 0.088, Pearson chi-square test). However, participants who knew that periodontal disease was a complication of DM (Q2) were more likely to know both the DM-to-periodontitis (Q4) and periodontitis-to-DM relationships (Q3) (*p* < 0.001 and *p* = 0.047 respectively, Fisher’s exact test). Again, no association was found between the awareness of either DM-to-periodontitis or periodontitis-to-DM relationship and participants’ demography or oral health behavior.

Concerning management of DM and periodontitis (Table 2), only about 2/3 participants knew the effect of periodontal treatment on DM (Q5). Nearly all participants knew the proper management of periodontal disease (regular dental checkup and proper daily oral hygiene) (Q6) [30]; and 2/3 participants knew that the correct approach of home periodontal care in DM patients involved both tooth brushing and use of dental floss/interdental brush (Q7) [31, 32]. It is also observed that less effective methods (toothpick and mouth wash) [33] were considered correct by a number of participants.

**Fig. 1:**
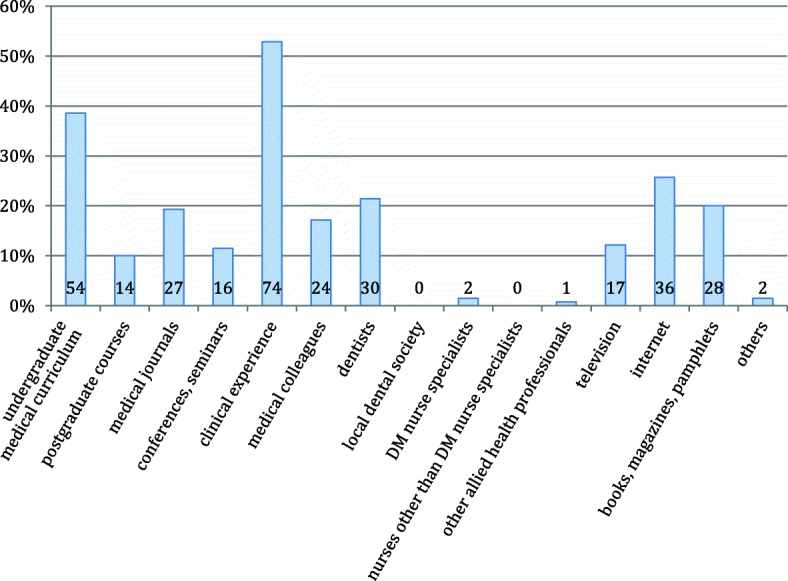
Source(s) of information concerning the relationship between periodontal disease and DM. Actual number is indicated on the bar

The information about the relationship between periodontal disease and DM came from very diverse sources (Fig. 1). The top five sources in descending order were: clinical experience (74/140), undergraduate curriculum (54/140), internet (36/140), dentists (30/140) and books, magazines and pamphlets (28/140).

Results on the relevant medical practice showed that: one fifth participants never asked DM patients for dental/gum problem and only 8 (5.7%) participants did it often (defined as ≥50% of cases); 18.6% participants never examined DM patients for oral/dental/gum condition and only 10 (7.1%) participants examined often; one fifth participants never recommended DM patients to see dentist, 17 (12.1%) participants recommended often. Among the 80% participants who did recommend their DM patients to see dentist, only 38.6% recommended specifically for periodontal screening.

**Fig. 2:**
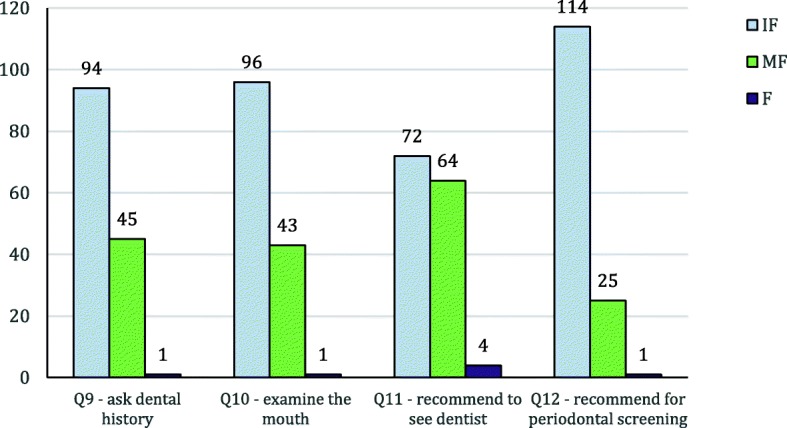
Results of Questions 9–12 after grouping. Grouping criteria: infrequent (IF) = (never + rarely), moderately frequent (MF) = (occasionally + sometimes), frequent (F) = (frequently + usually + every time)

Since the response to the practice questions were mostly in the low range (‘never’ to ‘occasionally’), they were grouped into three categories: infrequent (IF), moderately frequent (MF) and frequent (F) to facilitate further statistical analysis (Fig. 2):

The sample size of 140 did not have enough power for multivariate logistic regression which required at least 480 cases in the sample. Therefore, bivariate logistic regression was performed on the IF (as reference) and MF groups of Q9 to Q12 against individual demography, oral health behavior data and awareness data in Q1–7. The F group was ignored as the case number was too small. The results showed that clinical experience of the participants was significantly associated with asking dental history and doing oral examination. Practicing interdental cleaning was associated with recommending patients to see dentist. Clinical experience and being a FM specialist were associated with recommending patients to see dentist for periodontal evaluation (Table 3). However, none of the awareness factors in Q1–7 had significant association with the clinical practice.

**Table 3:** Factors associated with surveyed doctors’ clinical practice relating to periodontal health concern in DM patients

Crude odds ratio (95% CI‡)		*p*-value*
Factor associated with asking dental history (Q9)		
Clinical experience (per year)	1.069 (1.023–1.116)	0.003
Factor associated with doing oral examination (Q10)		
Clinical experience (per year)	1.059 (1.015–1.105)	0.008
Factors associated with recommending to see dentist (Q11**)**		
Practice interdental cleaning everyday	5.696 (1.157–28.035)	0.032
Practice interdental cleaning occasionally	5.500 (1.131–26.756)	0.035
Never practice interdental cleaning (reference)	1	
Factors associated with recommending to see dentist for periodontal evaluation (Q12)		
1. Clinical experience (per year)	1.054 (1.006–1.104)	0.027
2. FM specialist	4.000 (1.053–15.194)	0.042
Basic trainee (reference)	1	

‡*CI* confidence interval

*statistically significant taken as *p <* 0.05

Only factors with significant association were displayed. Insignificant factors (e.g. all awareness factors) were not shown

## Discussion

At least 10% Hong Kong population suffer from type 2 DM [23] and 40% people have some degree of periodontal disease [24]. With such high prevalence, it is expected that many people are suffering from both conditions. It is now well established that there is an interrelationship between them and such relationship appears to be bidirectional [14–16, 34]. Few studies were done to explore primary care doctors’ awareness of the interrelationship between DM, periodontal disease and their corresponding practice. One study in India surveyed medical interns on their awareness of periodontal disease in diabetic patients [35]. Another study in Jordan surveyed a mixture of primary care doctors and other specialists on this topic [36]. However, the authors did not consent to release their questionnaire on request and the questionnaire used was likely to be in their local language. Thus, a tailor made questionnaire was designed with the help of a periodontology professor and it has passed through the process of validation.

Majority of our participants were aware that there was a relationship between DM and periodontal disease. This knowledge was not affected by their age, years of experience, training status, demography or oral health behavior. They acquired the knowledge mostly from clinical experience and undergraduate medical curriculum (Fig. 1).

When we analyzed the awareness of the participants in more detail, it was found that there were significantly more people knowing the DM-to-periodontitis relationship than people knowing the periodontitis-to-DM relationship. There was also no association between the awareness of DM-to-periodontitis and periodontitis-to-DM relationships. Thus, it can be postulated that perhaps participants acquired the DM-periodontitis bidirectional knowledge separately from different sources or settings. It is also possible that whenever the relationship of DM and periodontal disease was discussed in literature, conference etc., the bidirectional relationship was inadequately emphasized. Furthermore, the DM-to-periodontitis relationship may be more frequently mentioned than the periodontitis-to-DM relationship in non-dental literature and conference.

The last part of the study aimed at exploring if awareness of the relationship between DM and periodontal disease turns into practice. There was big contrast between awareness and practice results. Although participants’ awareness of the various relationships between DM and periodontal disease was at least 60% (Q5 the lowest), only less than 10% participants often (defined as ≥50% of all DM patients) asked or examined their DM patients on dental problem and 12.1% recommended their DM patients to see dentist. Logistic regression showed that awareness had no effect on clinical practice though clinical experience, FM specialist status and personal interdental cleaning habit were associated with some periodontal health concern practice. The study in Jordan reported that 70% doctors knew the association between DM and oral health but only 53% recommended their DM patients to see dentist *regularly* [36]. In terms of awareness, doctors in Hong Kong seemed to be better than doctors in Jordan, but this was not observed in terms of recommendation. However, the study in Jordan comprised only 39.3% primary care doctors (others are endocrinologists, physicians and doctors of other specialties) from three different sources (university hospitals, private hospitals and community health clinics) and the exact percentage of *‘regular’* recommendation was not defined. Thus, results of the two studies can only be compared with caution. This high awareness but low recommendation phenomenon was also seen in another study in which 100% doctors were aware of a relationship between periodontal disease and general health but only 10% doctors referred their patients to dentists without patients asking for it [37].

It is noted that even endocrinologists, the expert in DM management may not recommend their patients to see dentist often. There was a study in China comparing endocrinologists and dentists on their awareness and practice regarding DM and periodontitis. In that study, only 26.6% endocrinologists reported that they frequently advised their DM patients to visit dentists [38]. In contrast, 61.2% dentists in this study reported frequent referral of their patients with severe periodontitis for DM evaluation.

In the Iranian hand hygiene study, poor hand hygiene practice among the residents was observed despite satisfactory knowledge on hand hygiene. The author attributed the reasons to time constraint and high workload of the residents [27]. In the current study, primary care doctors’ knowledge of the DM-periodontitis relationship also did not translate into practice. Thus their DM patients were unlikely to benefit much from their doctors’ knowledge. The reasons behind this phenomenon were not explored in this study but the predominantly private dental system in Hong Kong may be an obstacle for dental referral from public doctors. Even if a public doctor suggests a DM patient to see a private dentist, the patient may be reluctant to go in view of the cost. Another postulation is related to prioritization of problems and time constraint of doctors. DM patients usually have multiple medical co-morbidities such as hypertension and dyslipidemia, and the issue of polypharmacy is not uncommon. Apart from managing all these, doctors may also need to handle other episodic illness during follow up. Hence, managing periodontal disease may be put at lower priority within limited consultation time. Thus doctors in this study might have a common reason with the Iranian residents as far as poor translation of knowledge into practice was concerned. Other contributing factors warrant further research. Apart from enhancement on doctors’ practice, educating patients on the bidirectional relationship between DM and periodontal disease may also facilitate comprehensive management of both diseases.

The main limitation of this study was that only public doctors in one geographical location in Hong Kong were surveyed. The conclusion can only be applied to the Department of Family Medicine and Primary Health Care of KWC and cannot be generalized to public primary care doctors in other geographical locations or private primary care doctors in Hong Kong. A better study design can be achieved by random sampling all primary care doctors practicing in both private and public sectors so as to get more representative results. Including private doctors in the survey may be able to answer the question if dental referral rate from public doctors is significantly different from that of private doctors. Follow up questionnaires or qualitative studies could also be designed to explore more comprehensively the reasons why the surveyed primary care doctors did not recommend their DM patients to see dentists. Another limitation was that only bivariate instead of multivariate logistic regression was done. The latter required a sample even larger than our target population in order to analyze all the possible predictors simultaneously.

The survey was anonymous and included only doctors as study subjects. This was expected to minimize dishonesty in the answers. However, reporting bias could not be completely eliminated. Participants with unfavorable data (e.g. not aware of the relationship between DM and periodontal disease or not brushing teeth often) might have been more reluctant to return the questionnaire.

## Conclusions

A high proportion of the surveyed doctors in this study were aware of the relationship between diabetes and periodontal disease. More of them were aware of the DM-to-periodontitis relationship than the reverse periodontitis-to-DM relationship. Acquiring the knowledge regarding relationship between DM and periodontal disease may not benefit patients of the doctors concerned as the latter seldom appropriately put the knowledge into practice. Further measures to promote doctors’ practice and to enhance patients’ knowledge on DM and periodontal disease should be explored.

## Additional file

Additional file 1: Questionnaire on Diabetes Mellitus (DM) and periodontal disease: doctors’ awareness and practice. This is the questionnaire used in the study. (DOCX 112 kb)

## Abbreviations

DM: Diabetes mellitus
F: Frequent
FM: Family Medicine
GOPCs: General Out-Patient Clinics
HA: Hospital Authority
ICC: Intraclass correlation coefficient
IF: Infrequent
KWC: Kowloon West Cluster
MF: Moderately frequent
Q1, Q2, Q3 etc.: Question 1, 2, 3 etc.
SD: Standard deviation
SPSS: Statistical Package for the Social Sciences
