# Supplementary material for: Diabetes mellitus and periodontal disease: awareness and practice among doctors working in public general out-patient clinics in Kowloon West Cluster of Hong Kong
Source: BMC Fam Pract. 2018 Dec 17;19:199. doi: 10.1186/s12875-018-0887-2 (PMC6297978; doi:10.1186/s12875-018-0887-2)
Supplement: Supplementary file 1 — Questionnaire on Diabetes Mellitus (DM) and periodontal disease: doctors’ awareness and practice. This is the questionnaire used in the study. (DOCX 112 kb) [file 12875_2018_887_MOESM1_ESM.docx]

**Additional file 1: questionnaire**
